# Iatrogenic Type A Aortic Dissection Following Percutaneous Coronary Intervention

**DOI:** 10.7759/cureus.73884

**Published:** 2024-11-17

**Authors:** Hafiz Abdul Manan, Muhammad Khan, Muhammad Rafiq

**Affiliations:** 1 Cardiology, Darent Valley Hospital, Dartford, GBR; 2 Cardiology, Salisbury District Hospital, Salisbury, GBR; 3 Internal Medicine, St Johns Hospital, Livingston, GBR

**Keywords:** acute aortic dissection, anginal symptoms, iatrogenic complications, pci, percutaneous coronary intervention

## Abstract

Iatrogenic type A aortic dissection (IAAD) is a rare but life-threatening complication of percutaneous coronary intervention (PCI), often presenting significant therapeutic challenges. A looped guidewire between the right subclavian artery and brachiocephalic artery during coronary angiography (CAG) via right radial artery access can complicate catheter manipulation. This report discusses the case of a 58-year-old hypertensive female patient who developed aortic dissection during PCI, specifically due to wire manipulation at the brachiocephalic loop. The dissection extended retrogradely into the ascending aorta. Despite the severity, the patient remained hemodynamically stable and free from myocardial ischemia. Given her stability, conservative management focusing on blood pressure control was chosen. Serial CT imaging confirmed stabilization of the dissection. The patient underwent successful PCI via the femoral approach at a later date and was discharged the following day. This case underscores the potential for conservative management in select IAAD cases, emphasizing individualized treatment strategies.

## Introduction

Percutaneous intervention (PCI) is an effective treatment of angina/ischemic heart disease (IHD) in patients who suffer symptoms that are refractory to conservative medical therapy. The benefits of PCI must be carefully weighed against the risk of peri-procedural complications including bleeding, damage to blood vessels, introduction of infection, acute stroke, rupture of coronaries leading to pericardial effusion, cardiac tamponade, and acute aortic dissection [1].

Iatrogenic acute aortic dissection is a rare but potentially fatal complication during procedures like PCI and open-heart surgeries, including off-pump coronary artery bypass, thoracic aortic aneurysm repair, and transcatheter aortic valve replacement. It typically presents with sudden, severe chest or back pain and may lead to catastrophic complications such as aortic rupture, cardiac tamponade, or organ ischemia. Aortic dissections are divided into two types, A and B, based on the Stanford classification system depending on whether the ascending aorta is involved or not. Iotrogenic type A aortic dissection (IAAD) involves the ascending aorta and/or aortic arch, and possibly the descending aorta. This type is most likely hyperacute/acute in terms of presentation (onset less than 24 hours) and does require immediate surgical intervention in most cases. Type B dissection involves the descending aorta or the arch (distal to the left subclavian artery) and is treated medically as an initial treatment with surgery reserved for any complications. Aortic dissection is characterized by the tearing of the intimal layer of the vessel, creating a false lumen in between its different layers that can compromise blood flow to vital organs [2].

The inciting event can either be a direct injury of the sinus of Valsalva with the guiding catheter or dissection in the coronary artery that can extend retrogradely into the aortic root or antegrade extending forwards. Early recognition, prompt imaging, and appropriate surgical or medical management are crucial for survival. There is no established management strategy. However, optimal management, whether conservative or surgical, remains controversial, depending on clinical presentation and dissection extent. This case report aims to highlight the incidence of ascending aortic dissection during PCI and the management strategy used when this complication arose.

## Case presentation

**Figure 1:**
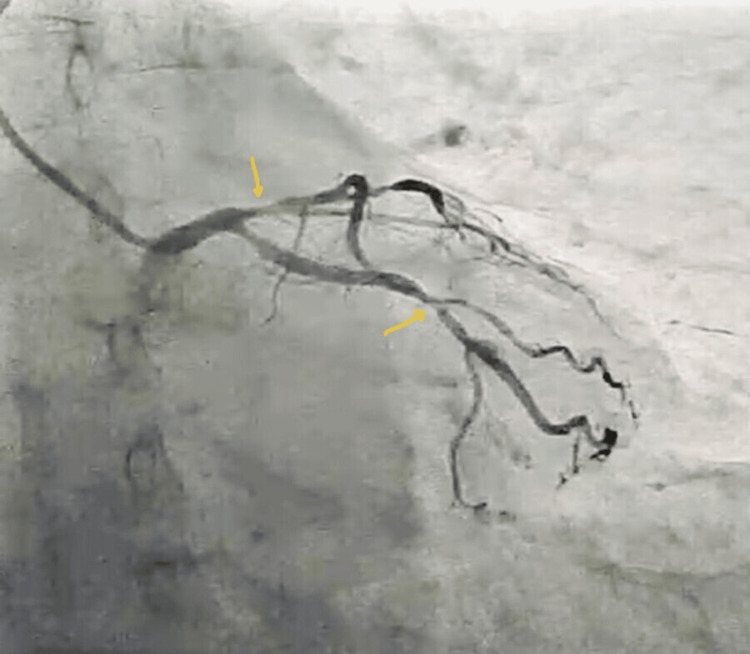
Coronary angiogram with arrows pointing at the proximal left anterior descending artery stenosis (90%) and moderate disease in left circumflex artery.

**Figure 2:**
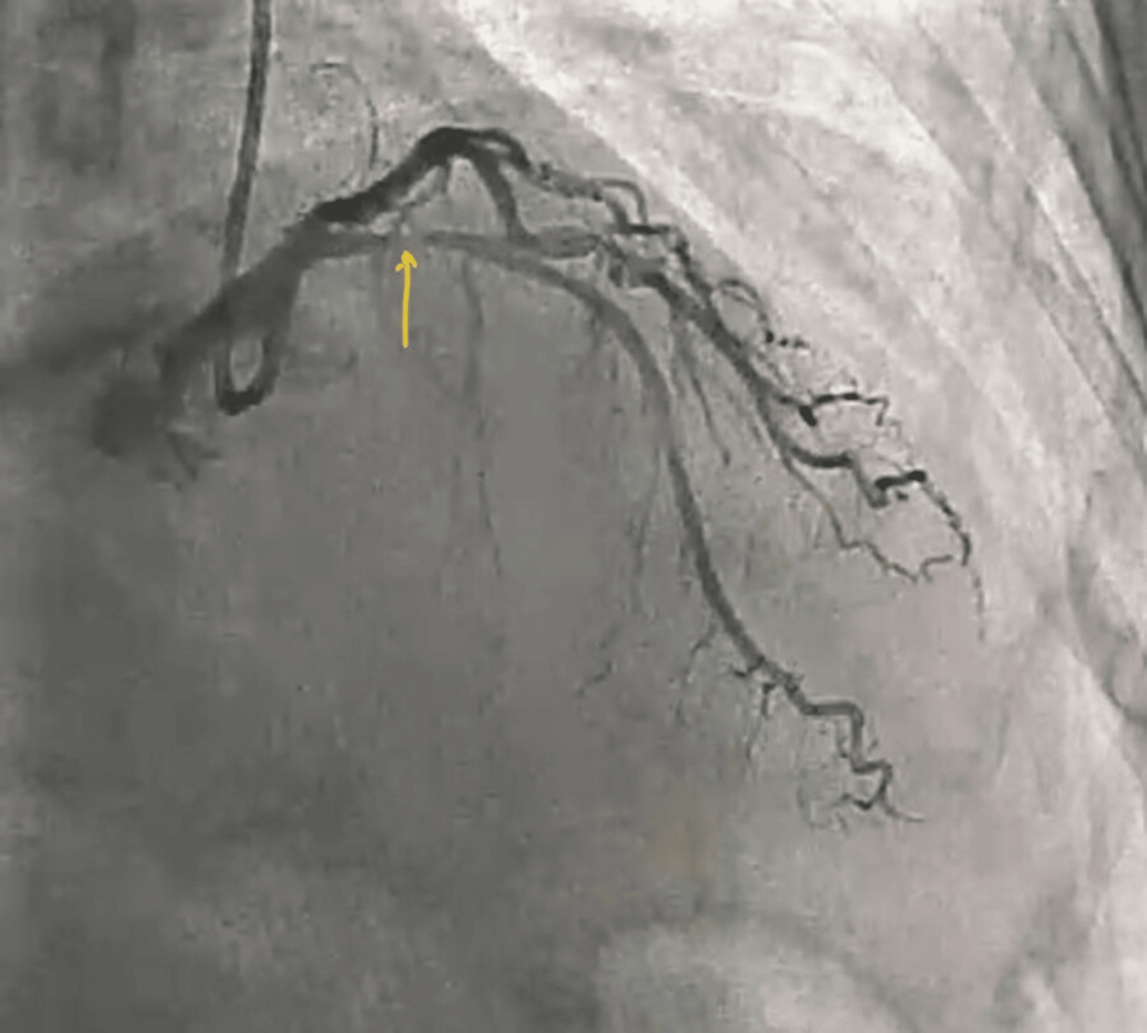
Tight stenosis (arrow) in proximal left anterior descending artery

**Figure 3:**
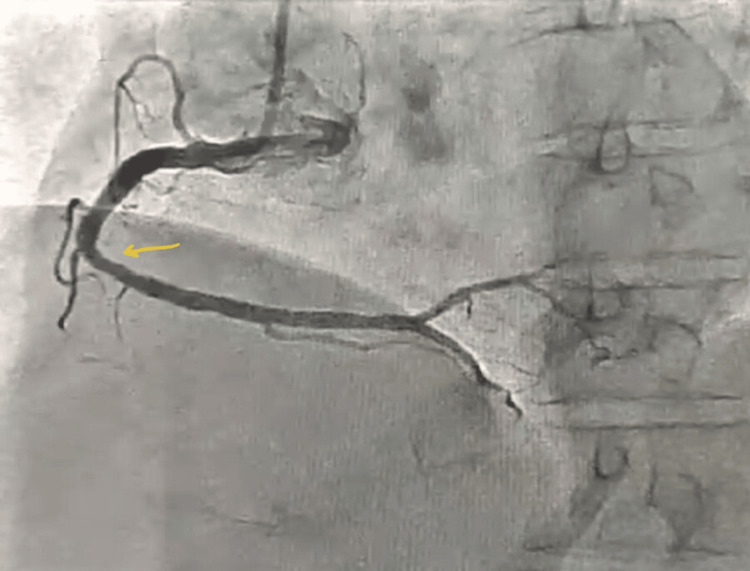
Mild disease (arrow) seen in right coronary artery

A 58-year-old hypertensive female patient presented with refractory anginal symptoms, partially relieved by anti-anginal medications. She had a history of poorly controlled hypertension due to non-compliance with medications. She was scheduled for outpatient coronary angiography. Coronary angiography via right radial artery access revealed significant stenosis in the proximal left anterior descending artery (LAD), moderate disease in the mid-left circumflex, and mild disease in a dominant right coronary artery (Figures 1-3).

**Figure 4:**
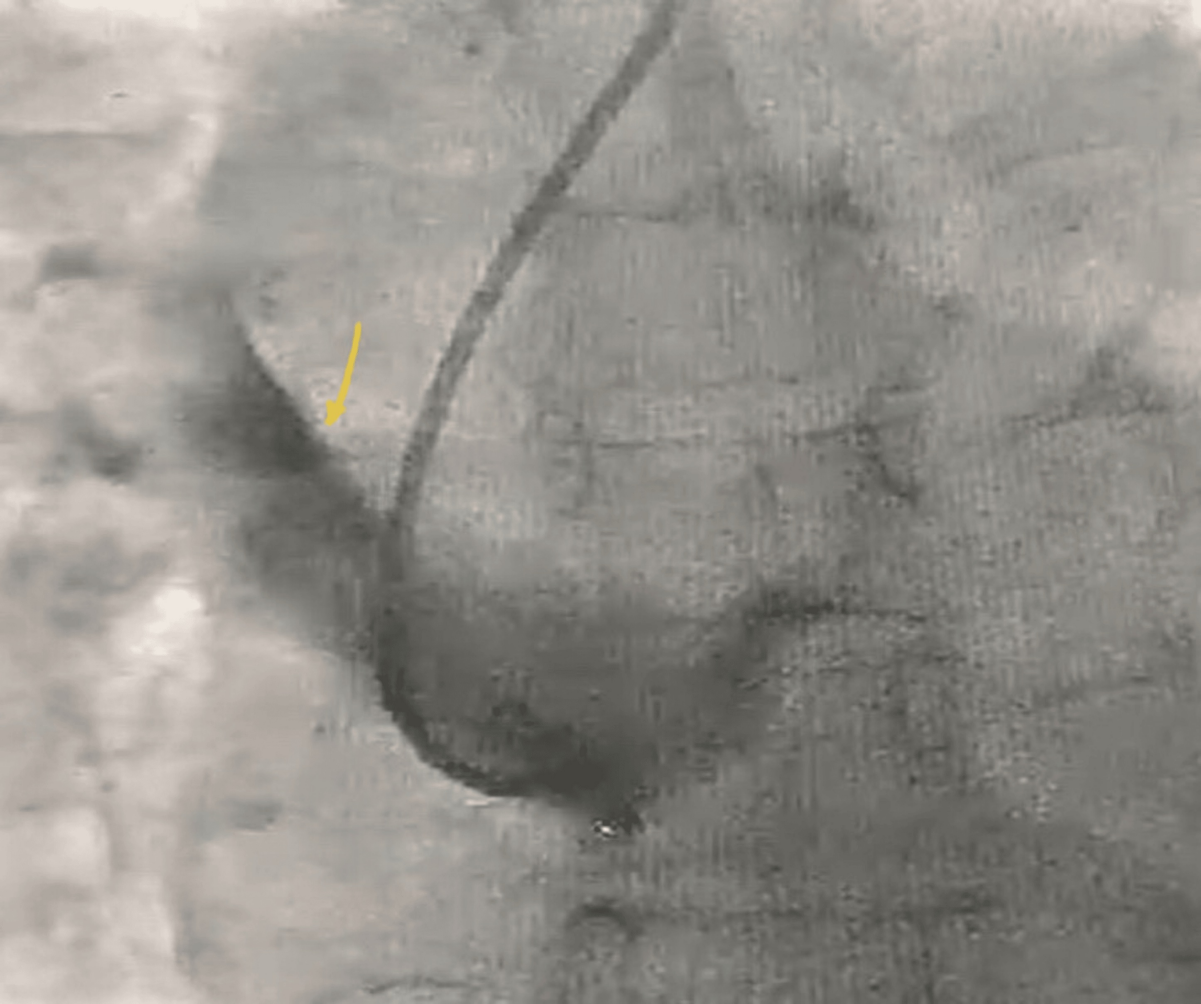
Guide catheter in ascending aorta showing ''type A ascending aorta dissection.'' Arrow indicates the contrast opacifying the ascending aorta

A looped guidewire at the brachiocephalic artery complicated the procedure. During wire manipulation at the brachiocephalic loop while preparing for PCI of the LAD, a type A aortic dissection occurred, extending retrogradely into the ascending aorta (Figure 4).

**Figure 5:**
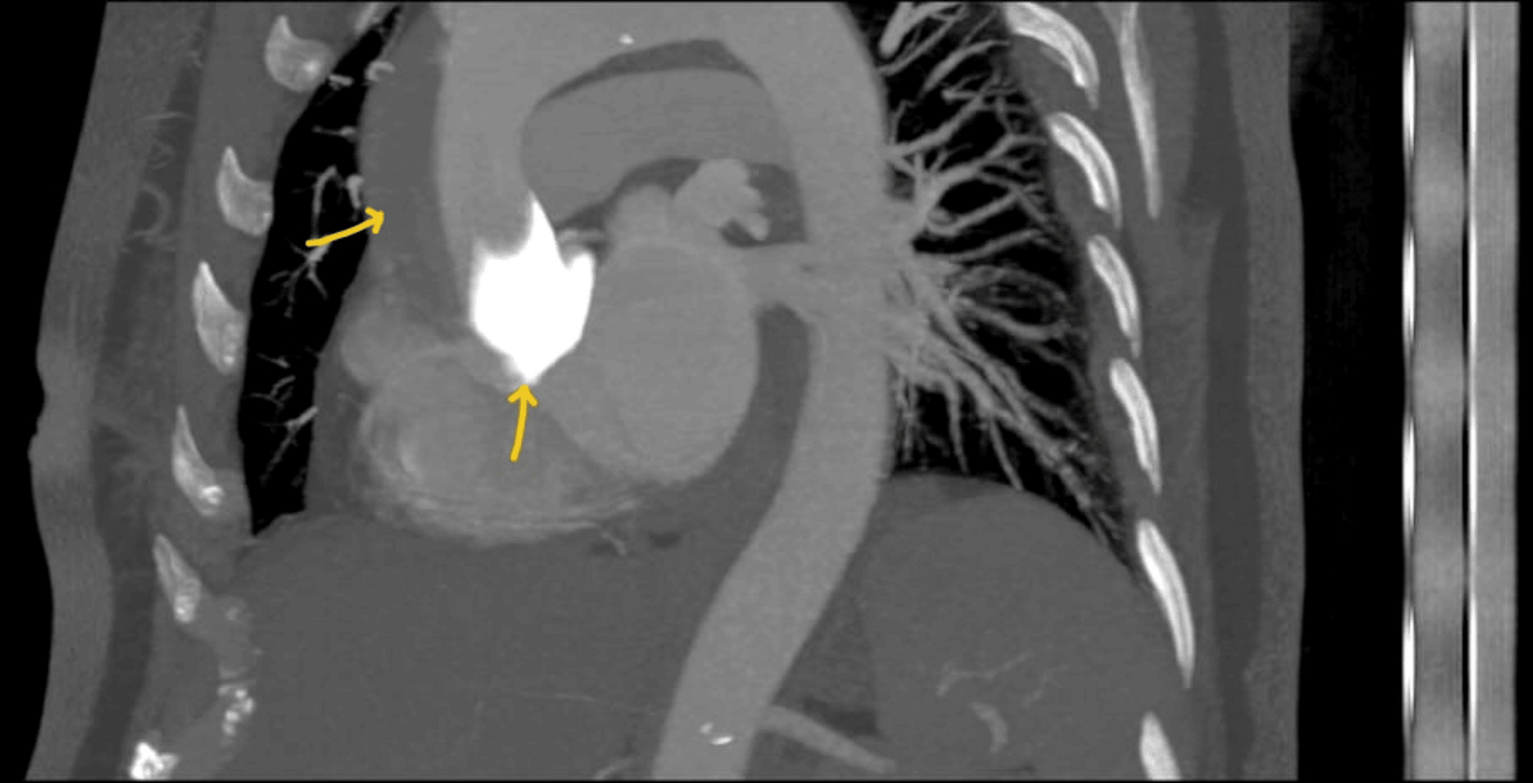
CT aortogram Arrow indicates the extravasation of contrast in dissection flap in the ascending aorta.

**Figure 6:**
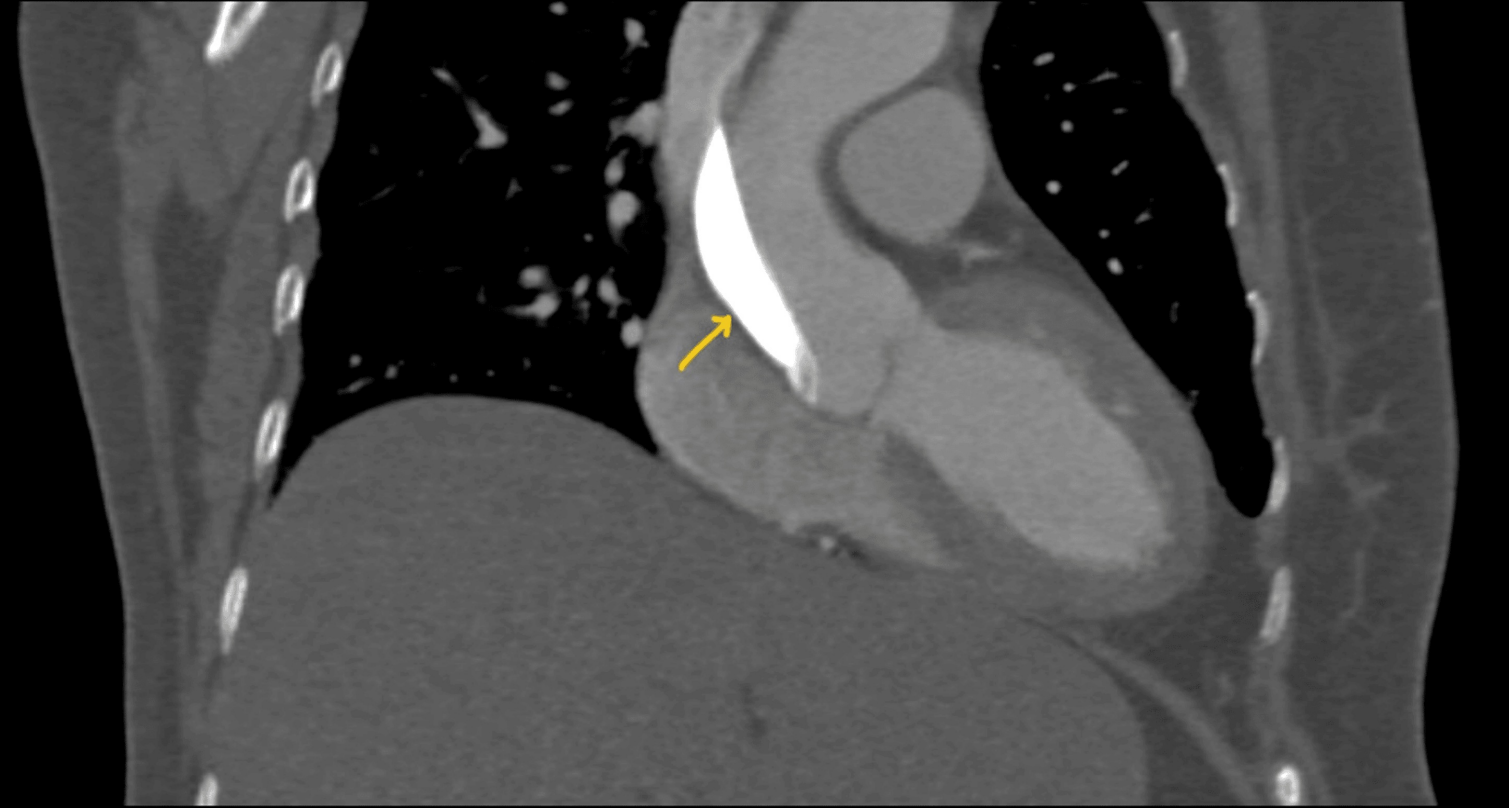
CT aortogram coronal section. Arrow indicates the further extension of contrast into dissection flap.

**Figure 7:**
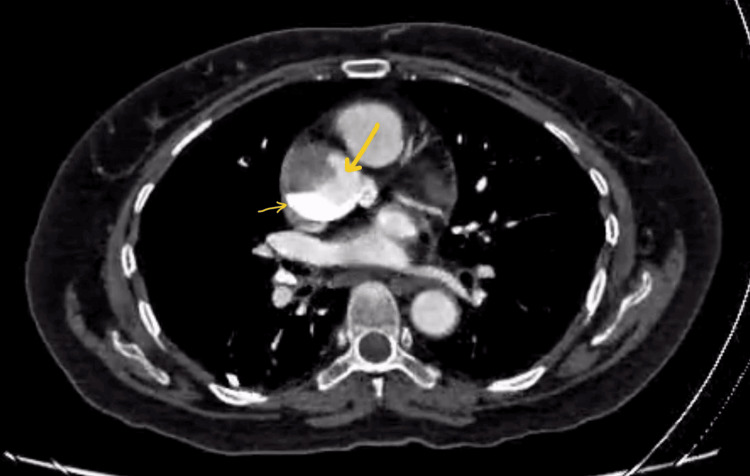
CT aortogram confirming ascending aortic dissection. Small arrow indicates the contrast in dissection flap; Bold arrow indicates the true lumen of ascending aorta.

Subsequently, an urgent CT aortogram confirmed the development of ascending aortic dissection (Figures 5-7).

The patient remained hemodynamically stable with mild throat discomfort. Echocardiography revealed no evidence of tamponade or involvement of the aortic valve. The cardiac surgeon on site was immediately consulted, patient was re-assessed by the surgical team and given the stable condition and no hemodynamic compromise, surgical intervention was deferred and conservative management with strict blood pressure control was initiated. The patient was admitted to the coronary care unit (CCU) for close monitoring.

Follow-up intervention

**Figure 8:**
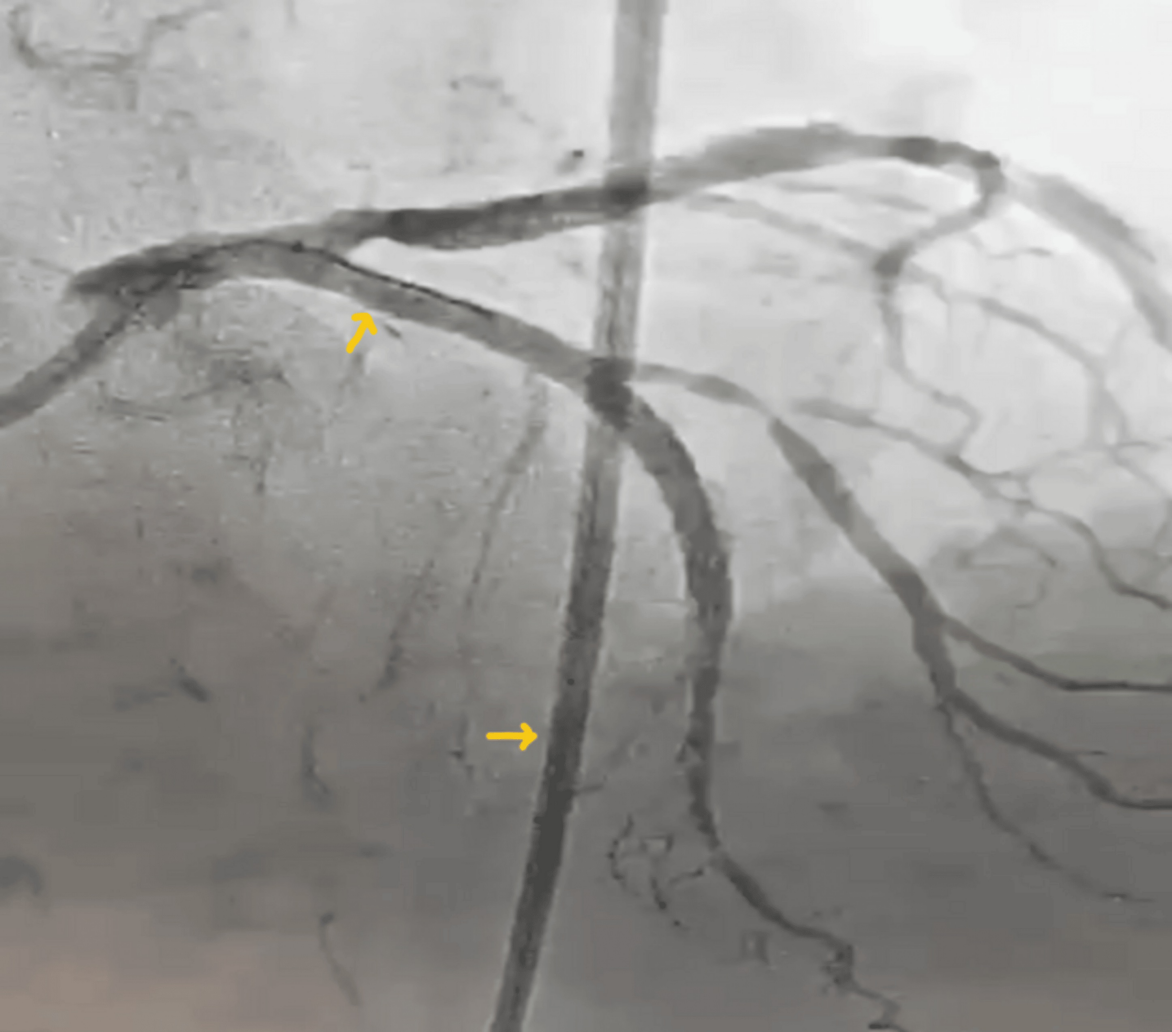
Coronary angioplasty showing the successfully placed stent in proximal LAD (vertical arrow) Horizontal arrow points at the guide catheter through femoral approach. LAD: left anterior descending artery

The patient later underwent successful PCI of the LAD via the femoral approach, with a stent deployed at the proximal-to-mid LAD (Figure 8).

**Figure 9:**
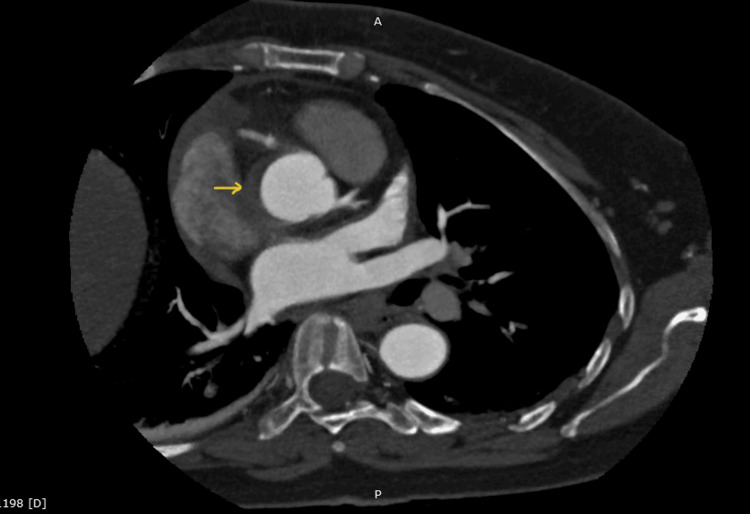
CT aortogram at the one-week follow-up showing the nearly resolved and stable dissection flap (arrow) with wide open true lumen of the ascending aorta

**Figure 10:**
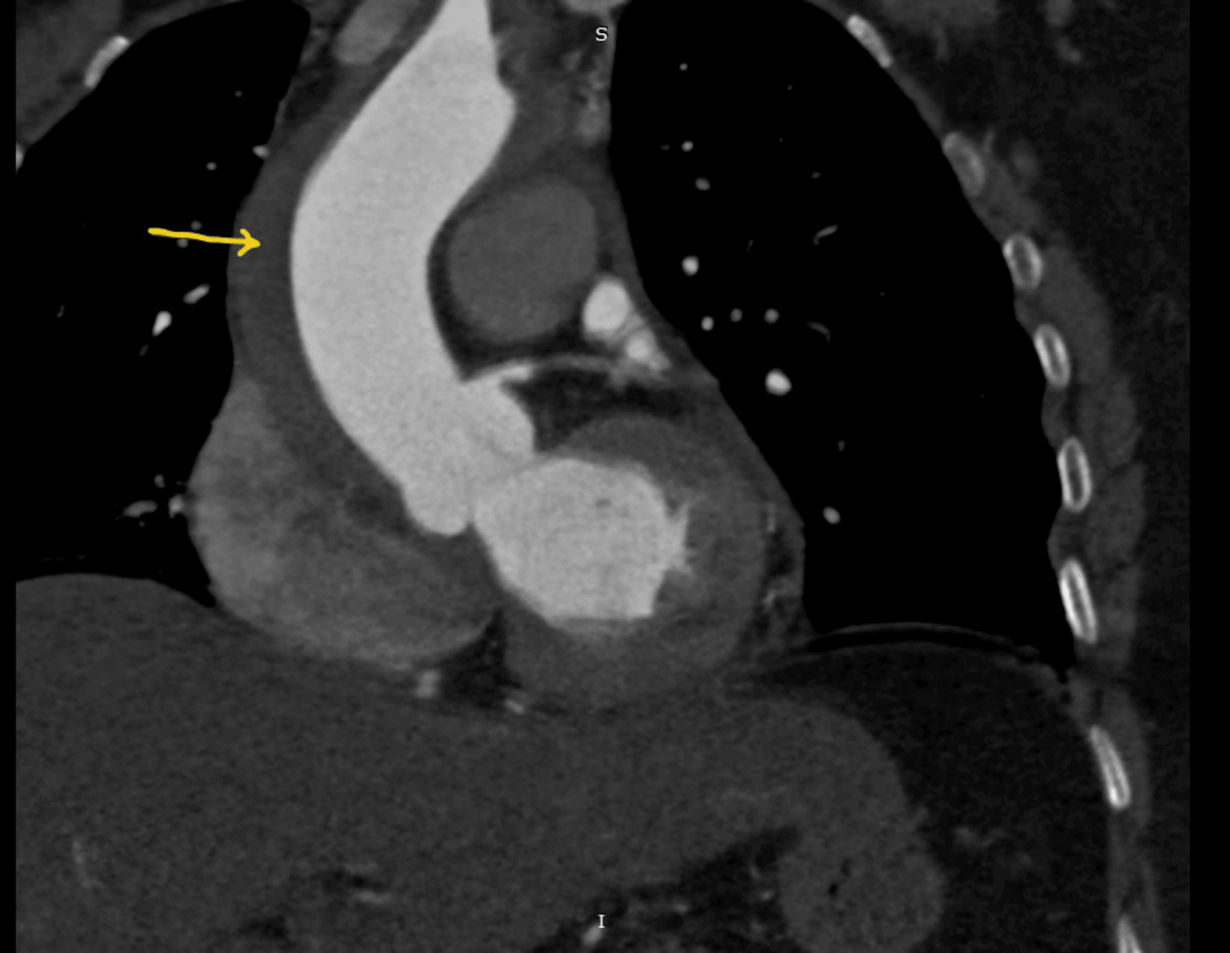
CT aortogram (coronal section) at the one-week follow-up showing the stabilized and significantly resolved dissection (arrow) in ascending aorta with no further extension or complications.

The patient recovered uneventfully and was discharged the next day totally asymptomatic. She was subsequently reviewed with another follow-up CT aortogram after one week showed stabilization and significant resolution of the dissection with no hemodynamic or clinical consequences (Figures 9, 10). The case report highlights the conservative approach taken to manage the IAAD.

## Discussion

Iatrogenic aortic dissections typically occur in the setting of complex cardiovascular conditions, such as chronic total occlusions, acute myocardial infarctions, or chronic heart failure due to valvular disease [1]. The pathogenesis involves an iatrogenic intimal tear with the blood leaking into a false lumen in between different layers of the vessel [2]. These cases often present as emergencies, with surgical management hindered by factors like continued anticoagulation, hyperglycemia, and other comorbidities. The incidence of catheter-induced aortic dissection during diagnostic and therapeutic coronary procedures is approximately 0.062% [3]. The diagnosis of IAAD during PCI is most commonly associated with symptoms of sharp, tearing chest pain which may be associated with a hemodynamic compromise [4]. However, in our case, the patient presented with mild throat discomfort, a less typical symptom that may warrant greater attention during coronary procedures [5]. The major risk factors for the development of aortic dissection are systemic hypertension, Marfan syndrome, and congenitally bicuspid or unicommissural aortic valves. Aortic medial degeneration has also been identified as a less important risk factor [6].

Diagnostic imaging is critical for IAAD diagnosis. Among the available established diagnostic procedures, CT aortogram has been traditionally the standard choice [7]. However, in some cases, it becomes challenging to diagnose dissection simply on contrast CT due to the complicated nature of dissection or the formation of hematoma in the dissection flap which makes the false lumen unclear [8]. Two-dimensional transthoracic color-flow Doppler echocardiography (TTE), transesophageal echocardiography (TEE), and magnetic resonance imaging (MRI) are recent advances in non-invasive imaging technologies that have been useful in assessing the thoracic aorta detailing the minute details like entry point, extent and exit point of dissection, and helping in devising the management strategy accordingly [9]. Laboratory markers like D-dimer and fibrin degradation products (FDP) can also provide evidence of acute aortic dissection [10].

IAAD is a rare complication that carries an excellent short- and long-term prognosis having possible options of surgical and conservative, nonsurgical approaches. When a coronary artery is involved as an entry point, it usually can be sealed safely with a stent with good results. However, in some cases, dissection could lead to acute aortic syndrome resulting in rapid clinical deterioration and hemodynamic instability, requiring urgent surgical intervention [11]. Conservative approaches such as blood pressure control and observation, are feasible for localized dissections that stabilize without further extension [12]. In our case, conservative management was appropriate due to the patient’s stable clinical condition and the absence of hemodynamic compromise [13,14].

## Conclusions

IAAD is a rare but serious complication of PCI. Early recognition, prompt imaging, and appropriate surgical or medical management are crucial for survival. Traditionally, symptomatic ascending aortic dissections had been, almost invariably, treated surgically. However, there are no clear management strategies for optimal management of relatively asymptomatic ascending dissections. It has been controversial whether the conservative or surgical approach is optimum depending on clinical presentation, location, and the extent of dissection. In some cases, urgent surgical intervention is needed; however, localized and non-complicated dissections where there is no evidence of ischemia or further extension of the dissection, can be managed safely by adopting conservative measures. Our case also demonstrates that conservative management can be a viable option in stable cases. Continued experience and research are needed to refine treatment guidelines for this rare complication.
